# Epigenetic and Transcriptional Modifications in Repetitive Elements in Petrol Station Workers Exposed to Benzene and MTBE

**DOI:** 10.3390/ijerph15040735

**Published:** 2018-04-12

**Authors:** Federica Rota, Anastasia Conti, Laura Campo, Chiara Favero, Laura Cantone, Valeria Motta, Elisa Polledri, Rosa Mercadante, Giorgio Dieci, Valentina Bollati, Silvia Fustinoni

**Affiliations:** 1 EPIGET, Epidemiology, Epigenetics and Toxicology Lab, Department of Clinical Sciences and Community Health, Università Degli Studi di Milano, via San Barnaba 8, 20122 Milan, Italy; rota.federica@gmail.com (F.R.); chiara.favero@unimi.it (C.F.); laura.cantone@unimi.it (L.C.); valeria.motta@unimi.it (V.M.); rosa.mercadante@unimi.it (R.M.); valentina.bollati@unimi.it (V.B.); 2 Department of Life Sciences, University of Parma, 43124 Parma, Italy; anastasia.conti@hotmail.it (A.C.); giorgio.dieci@unipr.it (G.D.); 3 Present address: San Raffaele Telethon Institute for Gene Therapy (SR-TIGET), 20132 Milan, Italy; 4 Occupational Medicine Unit, Fondazione Cà Granda, IRCCS Ospedale Maggiore Policlinico, 20122 Milan, Italy; laura.campo@policlinico.mi.it (L.C.); elisa.polledri@policlinico.mi.it (E.P.)

**Keywords:** benzene, petrol station workers, transcriptional activity of retrotransposons, methylation of DNA, methylation of histones

## Abstract

Benzene, a known human carcinogen, and methyl *tert*-butyl ether (MTBE), not classifiable as to its carcinogenicity, are fuel-related pollutants. This study investigated the effect of these chemicals on epigenetic and transcriptional alterations in DNA repetitive elements. In 89 petrol station workers and 90 non-occupationally exposed subjects the transcriptional activity of retrotransposons (LINE-1, Alu), the methylation on repeated-element DNA, and of H3K9 histone, were investigated in peripheral blood lymphocytes. Median work shift exposure to benzene and MTBE was 59 and 408 µg/m^3^ in petrol station workers, and 4 and 3.5 µg/m^3^, in controls. Urinary benzene (BEN-U), *S*-phenylmercapturic acid, and MTBE were significantly higher in workers than in controls, while *trans,trans*-muconic acid (*tt*-MA) was comparable between the two groups. Increased BEN-U was associated with increased *Alu-Y* and *Alu-J* expression; moreover, increased *tt*-MA was associated with increased *Alu-Y* and *Alu-J* and LINE-1 (L1)-5′UTR expression. Among repetitive element methylation, only L1-Pa5 was hypomethylated in petrol station workers compared to controls. While L1-Ta and Alu-YD6 methylation was not associated with benzene exposure, a negative association with urinary MTBE was observed. The methylation status of histone H3K9 was not associated with either benzene or MTBE exposure. Overall, these findings only partially support previous observations linking benzene exposure with global DNA hypomethylation.

## 1. Introduction

Petrol vapors and traffic emissions contain high levels of toxic compounds, which might be a relevant exposure in particular occupationally-exposed individuals, such as traffic policemen and petrol station workers [1,2,3].

The International Agency for Research on Cancer (IARC) has classified both petrol and petrol engine exhaust as possibly carcinogenic to humans (group 2B) [4]. Among petrol components, benzene is of particular concern as it is a known carcinogen for humans (group 1) [5]. MTBE, however, is not classifiable as to its carcinogenicity to humans (group 3) [6].

In our previous studies on traffic policemen and petrol station attendants, exposure to traffic emissions and petrol vapors was assessed by environmental and biological monitoring, the latter using urinary benzene and MTBE [1,2,7]. These studies suggested that urinary benzene is a good biomarker for benzene exposure, although impacted by smoking; on the other hand, urinary, MTBE is a specific biomarker of traffic and petrol vapors, and is not influenced by smoking.

Recent evidence suggests a possible role for epigenetic changes in benzene-induced leukemogenesis. In particular, our research group has demonstrated dose-dependent deregulation of human DNA methylation in traffic policemen and petrol station attendants exposed to low levels of benzene [8,9], with an exposure-related decrease in global DNA methylation and in LINE-1 (L1) and Alu retroelement methylation, suggesting that retroelement hypomethylation could be among the factors contributing to benzene hematotoxicity. Despite the mentioned data, the evidences in vivo are still limited and mainly reporting associations more than a causal link between exposure, epigenetic variations and cancer development, which would request the use of prospective studies in order to avoid reverse causation. The link between hypomethylation and benzene exposure was further supported by an in vitro study [10], showing global DNA hypomethylation in human TK6 lymphoblastoid cells treated with hydroquinone, an oxidated metabolite of benzene. Subsequently, benzene was also shown to impact CpG methylation of specific genes in in vitro [11,12] and in vivo studies [10,13,14]. Because the CpG content and DNA methylation levels of repetitive elements vary dramatically across subfamilies with different evolutionary age, repetitive element subfamilies may also have differential sensitivity to environmental exposures [15].

The epigenetic silencing of retroelement activity through DNA methylation is thought to be an important cellular defense mechanism to repress retroelement amplification, which threatens genome stability [16]. Therefore, global loss of methylation at Alu and L1, which together account for a significant fraction of all potential methylation sites (CpG dinucleotides) in the human genome, may result in genome-wide transcriptional de-repression, which, in turn, may have a number of consequences at both the RNA and chromatin level [17,18,19]. In principle, Alu and L1 RNA upregulation could either promote or counteract pathogenesis. For example, overexpression of an Alu-like RNA has been shown to promote cell differentiation and to reduce the malignancy of human neuroblastoma cells [20]. On the other hand, changes in L1 and Alu promoter methylation between normal and cancer cells have been reported, with a clear tendency toward hypomethylation in malignant transformation [21]. Recently, histone H3K9 methylation has been proposed as the primary mechanism for suppressing the transcription of Alu elements [22]. Moreover, we have recently reported in vitro evidence for retroelement transcriptional derepression in response to hydroquinone even in the absence of DNA hypomethylation [23] highlighting the complexity of retroelement regulation.

With the aim of elucidating the mechanism of benzene toxicity, the present study investigated the effects of petrol vapor exposure, particularly benzene and MTBE exposure, on petrol station workers. The petrol station workers and control subjects frequency-matched for general characteristics and smoking habits (*N* = 90) were subjected to the evaluation of the following parameters in peripheral blood mononuclear cells: (i) repetitive elements expression (in particular, Alu-Y, Alu-J, L1-5′UTR, and L1-ORF1); (ii) the degree of methylation of different repetitive element subfamilies with different evolutionary age (in particular, Alu-Yb8, Alu-YD6, Alu-SX, L1-HS, L1-Pa2, L1-Pa5, and L1-Ta); and (iii) global determination of histone H3K9 methylation.

## 2. Materials and Methods

### 2.1. Study Subjects and Sample Collection

The study included healthy male adults (≥18 years old): 89 petrol station workers exposed and 90 non-occupationally exposed subjects (controls), all working and living in the North-West area of the Milan province, within a 25 km distance from Milan city center. Among control subjects, mostly worked outdoor and were involved in maintenance activities (bricklayers, carpenters, plumbers, electricians); they were individually-matched by gender, age, body mass index (BMI), and smoking habits with workers. The subjects’ personal and occupational characteristics, including health status, drug assumption, smoking habits, use of products containing solvents in the last 24 h, means of transport and time to get to job place, were collected by a questionnaire administered by trained interviewers. No occupational exposure to diesel or exhaust fumes from vehicles, nor the use of solvents/products containing chemicals of concern was reported by control subjects (for details, see reference [1]).

The subjects were tested for personal exposure to airborne benzene (BEN-A) and methyl *tert*-butyl ether (MTBE-A), for a 5-h work-day period (beginning 6:00–9:00, ending 11:00–14:00), in the second part of the work week, typically on Thursday. Three urine samples were collected from each subject on Monday morning, before the beginning of the study week (baseline), and before and at the end of the investigated work shift. Urine samples were analyzed for urinary benzene (BEN-U), *trans,trans*-muconic acid (*tt*-MA), *S*-phenylmercapturic acid (SPMA), and urinary MTBE (MTBE-U) biomarkers. To avoid the interference of diet on *tt*-MA, subjects were requested to refrain from food containing sorbic acid and sorbates as preservative the day before and the day of urine sampling. Air and urine sample collection and treatment were conducted as described previously [1].

The morning after the air exposure assessment two aliquots of whole blood were collected by each subject by venous phlebotomy. One aliquot (about 5 mL blood) was used for the determination of blood cell count, liver enzymes (aspartate transaminase, AST, alanine transaminase, ALT, and gamma-glutamyltransferase, GGT), lipidic profile (cholesterol, triglycerides, high-density lipoprotein, HDL, and low-density lipoprotein, LDL), and the inflammatory marker C reactive protein (CRP); measurements were performed by a chemico-clinical laboratory using routine assays. The second aliquot (about 7 mL blood into an EDTA tube) was used for the determination of epigenetic and transcriptional markers; with this aim each sample was coded (blind to the experimenters) and delivered to the laboratory within 3 h using thermal bags to prevent sample exposure to extreme temperatures. The study was conducted in the frame of the workers’ periodical health surveillance according to Italian law DLgs 81/08. Written informed consent was obtained from each of the study subjects.

### 2.2. Air and Urinary Exposure Assessment

Individual personal exposure was monitored with a passive air sampler (Radiello, Sigma Aldrich, Milano, Italy) equipped with a 35–50 mesh charcoal cartridge (Supelco, Sigma-Aldrich, Milan, Italy) worn by subjects in the respiratory zone. The determination of BEN-A and MTBE-A was performed by gas chromatography mass spectrometry (GC/MS), the determination of BEN-U and MTBE-U was performed by headspace solid phase microextraction followed by GC/MS, and the determination of *tt*-MA and SPMA was performed by LC-MS/MS [1]. The limit of quantification (LOQ) was 1.0 µg/m^3^ for BEN-A and 0.8 µg/m^3^ for MTBE-A, 15 ng/L for BEN-U and 10 ng/L for MTBE-U, 20 µg/L for *tt*-MA and 0.1 µg/L for SPMA. Levels of urinary cotinine (COT-U), a biomarker of tobacco smoking, were measured by LC-MS/MS, with a LOQ of 1 µg/L [24]. Subjects with COT-U concentrations below 100 µg/L were classified as “non-smokers”, while those with COT-U concentrations equal to or above 100 µg/L were classified as “smokers”. Urinary creatinine was determined by Jaffe’s colorimetric method [25]; urine sample were considered valid when creatinine was in the range 0.3–3 g/L.

### 2.3. DNA, RNA, and Histone Purification

Blood cell counts were performed on fresh blood by automated methods (Coulter Cell Counter, Abbott or Bayer) within 3 h of the blood draw. Blood was centrifuged at 2500 rpm for 15 min. The buffy coat fraction was transferred to a cryovial and immediately frozen at −80 °C until use. In addition, whole blood was stabilized in PAXgene Blood RNA tubes (Qiagen–PreAnalytix, Hombrechtikon, Switzerland), kept at room temperature for 24 h and then stored in a freezer (−20 °C) until RNA extraction. Total RNA was isolated using the MagMAX-96 Total RNA Isolation Kit (Life Technologies) and RNA quality was assessed by a 2100 Agilent Bioanalyzer (Agilent Technologies, Inc., Waldbronn, Germany). Minimum quality was defined as A260/A280 > 1.9, A230/A280 > 2 and an RNA integrity number >8. Genomic DNA was extracted from frozen buffy coat using the Wizard Genomic DNA purification kit (Promega, Madison, WI, USA) according to the manufacturer’s instructions. DNA concentration was calculated spectrophotometrically at 260 nm on a NanoDrop ND-1000 Spectrophotometer V3.7 (Thermo Scientific, Rodano, Italy). Extracted DNA was aliquoted and stored at −80 °C.

To extract the histone fraction, red blood cell lysis solution (Promega) was added to the buffy coat to lyse red blood cells. After a 10-min incubation at room temperature, the mixture was centrifuged at 2500× *g* for 15 min, and the supernatant was discarded. Remaining monolayer cells were processed according to the protocol used by Chen et al. [26]. Briefly, cells were lysed in 1 mL ice-cold radioimmunoprecipitation assay buffer (Santa Cruz Biotechnology, Santa Cruz, CA, USA) supplemented with a protease inhibitor mixture (Roche Applied Sciences, Indianapolis, IN, USA) for 10 min. The sample was then collected and centrifuged at 10,000× *g* for 10 min. After discarding the supernatant, the remaining pellet was re-suspended in 0.4 N H_2_SO_4_. After incubation on ice for 90 min, the sample was centrifuged at 14,000× *g* for 15 min. The supernatant was mixed with cold acetone and stored at −20 °C overnight. The precipitated histones were collected by centrifugation at 14,000× *g* for 15 min. After one wash with acetone, the histones were air dried and resuspended in 500 μL water. We measured total protein in each sample by the Bradford assay according to manufacturer’s instructions (protein assay kit 500-0002; Bio-Rad Laboratories, Milan, Italy). We used equal amounts of total protein (4 μg) to normalize the results of the subsequent analysis on histones.

### 2.4. Repetitive Element Expression

cDNA was synthesized from 500 ng of total RNA using an iScript cDNA Synthesis Kit (Bio-RAD). Expression was analyzed by real-time PCR using an AB7300 Real-Time PCR System (Applied Biosystems, Foster City, CA, USA). Primers were designed to amplify members of the Alu-S subfamily (Alu-Y pair), Alu-J subfamily (Alu-J pair) and either the 5′UTR or the ORF1 regions of L1-Hs and L1-PA2 subfamilies. L1-5′UTR primers were able to detect full-length transcripts, while L1-ORF1 primers detected both full-length and 5′-truncated elements. Primer sequences are reported in Supplementary Figure S1. All measurements were normalized to the expression of B2M (β2-microglobulin housekeeping gene) and all PCR runs were performed in technical triplicate. Gene expression levels for each target were expressed as ΔCT, defined as the cycle threshold (CT) value for the target sequence minus the CT for B2M (CTsequence − CTB2M = ΔCTsample).

### 2.5. DNA Methylation Analysis

One-microgram aliquots of DNA (concentration 50 ng/µL) were treated with bisulfite by application of the EZ DNA Methylation-Gold™ Kit (Zymo Research, Orange, CA, USA) according to the manufacturer’s protocol. The final elution was performed with 30 µL of M-Elution Buffer. Bisulfite-treated DNA was stored at −20 °C and used shortly after treatment. Analysis of DNA methylation was performed using previously published methods [15] with minor modifications. Briefly, a 50-µL PCR was carried out with 25 µL of GoTaq Green Master mix (Promega), 1 pmol of the forward primer, 1 pmol of the biotinylated reverse primer, 50 ng of bisulfite-treated genomic DNA and water. Primer sequences and PCR conditions are reported in Supplementary Figure S1. The PCR products were purified by binding to Streptavidin Sepharose HP beads (Amersham Biosciences, Uppsala, Sweden) washing, denaturing to single stranded DNA using a 0.2 M NaOH solution, and washing again using the Pyrosequencing Vacuum Prep Tool (Pyrosequencing, Inc., Westborough, MA, USA), as recommended by the manufacturer. Then, 0.3 µΜ pyrosequencing primer was annealed to the purified single-stranded PCR product and pyrosequencing was performed using the PyroMark MD System (Pyrosequencing, Qiagen, Germantown, MD, USA). The degree of methylation was expressed as a percentage of methylated cytosines divided by the sum of methylated and unmethylated cytosines (%5mC). Measurements for each subject were run in duplicate.

### 2.6. Histone-3-lysine-9 Trimethylation (H3K9me3)

We used a solid-phase sandwich enzyme-linked immunosorbent assay (ELISA) with monoclonal antibodies to detect endogenous levels of H3K9me3 (Epiquik Global Tri-Methylation Histone H3-K9 Quantification Kit, cat. no. P-3034, Epigentek, Brooklyn, NY, USA) according to the manufacturer’s protocol. We used a Synergy HT-BioTek spectrophotometer to read the optical density at 490 nm, which was assumed proportional to the concentration of modified histones. Every sample was tested two times for each assay to increase reliability. The average of the two runs was used in the statistical analysis.

### 2.7. Statistical Analysis

All statistical analyses were performed with SAS software, version 9.3 (SAS institute, Cary, NC, USA). For markers of benzene and MTBE exposure, a value corresponding to one-half of the quantification limit was assigned to measurements below analytical quantification. General group characteristics were compared by chi-square test, *t*-test, or non-parametric Wilcoxon rank-sum test for non-normally distributed variables. Continuous variables are expressed as mean ± SD or as median (10th–90th percentile) as appropriate; for exposure parameters also minimum and maximum are given. Categorical variables are presented as absolute numbers and frequencies.

Multivariable linear regression models were applied to verify associations between petrol station workers/controls status and gene expression, between airborne exposure levels and gene expression, and also between urinary biomarkers (creatinine-adjusted) and gene expression. These models were used with the same independent variables to examine associations with histone modification. H3K9me3 concentrations were log-transformed to achieve a normal distribution.

Linear mixed-effect models were fitted to estimate differences between petrol station attendants and controls on DNA methylation and to estimate the effects of airborne exposures and urinary biomarkers (creatinine-adjusted) on DNA methylation. The pyrosequencing-based DNA methylation analysis tests a variable number of CpG positions, according to CpG density in the promoter being tested. A linear mixed-effect model was used to account for each CpG dinucleotide position measured in each duplicate and the potential confounding effect of the plate. An unstructured covariance matrix structure was used to model within-subject errors. The Kenward-Roger approximation was used to estimate the degrees of freedom in the denominator. DNA methylation was treated as a continuous variable, and the CpG site was considered a random effect.

In models including urinary biomarkers as covariates, biomarkers were introduced, at first, as levels at baseline, before and at the end of the investigated work shift, separately; then the geometric mean value of the three sampling moments was calculated and introduced in the model to better represent the overall individual body burden [27]. As no relevant difference was found between these approaches, the model with the geometric mean value of multiple determinations was finally adopted. All models described above were adjusted for age, BMI, cotinine (as a metric of tobacco smoking), and lymphocyte percentage. Linear regression models for H3K9me3 analysis were adjusted using a standard curve. The roles of other possible covariates, among which results of blood test, were evaluated through univariate analyses, but none of the covariates resulted in an association with gene expression, DNA methylation or histone modification. We calculated standardized beta and Δ% coefficients and 95% confidence intervals (CIs) that express the change in gene expression, DNA methylation and histone modification, respectively, associated with an increase in airborne or urinary exposure equal to the difference between the 90th and 10th percentile of the exposure distribution. A *p*-value ≤0.05 was considered statistically significant.

## 3. Results

### 3.1. Study Subjects and Exposure Assessment

A summary of personal characteristics, smoking habit, urinary creatinine, and personal exposure to benzene and MTBE of the study subjects, as assessed by air sampling and urinary biomonitoring, is reported in Table 1. Based on creatinine level, all urine samples were considered valid and retained for further analysis. Petrol station workers and controls were matched for gender (all males), age, BMI, and smoking status. Airborne exposure was significantly higher in petrol station workers than in controls, with median levels of BEN-A of 59 vs. 4 µg/m^3^ and of MTBE-A of 408 vs. 3.5 µg/m^3^, respectively. Levels of the urinary biomarkers BEN-U, SPMA, and MTBE-U were significantly higher in petrol station workers than in controls, whereas *tt*-MA levels were similar between the two groups. The influence of diet on *tt*-MA was negligible, given the good adherence of study subjects to diet restriction to avoid food containing sorbic acid and sorbates. A summary of health status, including work-related symptoms, present and previous diseases, drug assumption, blood cell count, liver enzymes, triglycerides, cholesterol and C-reactive protein (CRP) in petrol station workers and control subjects, are presented in Supplementary Table S1.

### 3.2. Repetitive-Element Expression, Repetitive-Element Methylation, and H3K9me3 Modification by Occupational Groups

The results of our comparisons of repetitive element expression (Alu-Y, Alu-J, L1-5′UTR, L1-ORF1), repetitive element methylation (Alu-YB8, Alu-YD6, Alu-SX, L1-HS, L1-Pa2, L1-Pa5, L1-Ta), and H3K9me3 histone modification between petrol station attendants and referents are reported in Table 2. Notably, we observed significant L1Pa5 hypomethylation in petrol station attendants compared to control subjects (β = −1.02; *p* = 0.05). There were no differences in expression or methylation of the other markers between the two groups.

### 3.3. Repetitive Element Expression in Relation to Exposure Assessment

Expression of repetitive elements (Alu-Y, Alu-J, L1-5′UTR, L1-ORF1) was not associated with exposure to airborne benzene and MTBE (Table 3). In contrast, we observed a significant association between repetitive element expression and BEN-U and *tt*-MA levels. In particular, as reported in Table 4, we observed an increased expression of Alu-Y and Alu-J in association with BEN-U (β_90-10_ = 0.290, *p* = 0.021 and β_90-10_ = 0.458, *p* = 0.001, respectively). We also observed an increased expression of Alu-Y, Alu-J and L1-5′UTR in association with *tt*-MA (β_90-10_ = 0.410, *p* = 0.0003, β_90-10_ = 0.331, *p* = 0.0100, and β_90-10_ = 0.27, *p* = 0.0070, respectively). β_90-10_ expresses the change in methylation (%5mC) for an increase equal to the difference between the 90th and 10th percentile of the urinary metabolite distribution, whereas not a standardized β coefficient measures the change in outcome variable for one unit increase in a predictor variable.

### 3.4. Repetitive Element Methylation and H3K9me3 Modification in Relation to Exposure Assessment

Differences in percent methylation among different repetitive element subfamilies was not associated with airborne benzene or MTBE exposure levels (Table 3). Table 4 reports the associations between the percent methylation in repetitive element subfamilies and urinary metabolite concentrations. The percent methylation of L1-HS was inversely associated with *tt*-MA concentration (β_90-10_ = −0.515; *p* = 0.0620); moreover, a tendency toward hypomethylation was also apparent for the other repetitive elements, although these associations were not significant. In a linear regression model, Alu-YD6 methylation was inversely associated with the MTBE-U concentration (β_90-10_ = −0.257; *p* = 0.0411). A similar behavior was observed for L1-Ta (β_90-10_ = −0.279; *p* = 0.0298). The estimated changes in DNA methylation after adjustment for age, BMI, cotinine levels and percent lymphocytes did not differ from the crude estimates (data not shown). H3K9 methylation was not associated with airborne benzene levels (Table 3) nor with urinary metabolites (Table 4).

## 4. Discussion

In this study, we investigated the molecular changes occurring at retroelements at three different levels: RNA expression, DNA methylation, and H3K9 methylation in petrol station workers and in non-occupationally exposed subjects. Increased levels of benzene biomarkers were associated with increased Alu-Y and Alu-J expression (BEN-U and *t,t*-MA), and LINE-1 (L1)-5′UTR expression (*t,t*-MA). Only the subfamily L1-Pa5 was hypomethylated in petrol station workers compared to controls, while L1-Ta and Alu-YD6 methylation was negatively associated with MTBE-U, but not with benzene biomarkers. Conversely, other retroelements expression and methylation, or the methylation of histone H3K9 were not associated with either benzene or MTBE exposure.

The main hypothesis of our study was that occupational exposure to benzene might be associated to molecular events occurring at retroelements, which are relevant as they might potentially play a key role in early leukemogenesis. In particular, L1 and Alu promoters could be considered highly sensitive switches interspersed throughout the genome; normally found in a repressed state, they could be switched on in response to environmental stimuli, ultimately inducing alterations to genome expression and stability, thus favoring cancer development. Previous studies conducted by our research group and by others revealed both global and gene-specific methylation reduction in subjects who were occupationally exposed to benzene [8,9,28], suggesting that epigenetic switching at retroelements might contribute to carcinogenicity [29].

Alu elements are the most abundant repeated elements in the human genome; with more than a million copies, representing 10% of the total genome [30]. Alu elements are divided into three major subfamilies known as Alu-J and Alu-S and Alu-Y. The Alu-Y subfamily, the most evolutionarily recent subfamily, is the only retrotranspositionally active, and is the only one that contributes to genome instability [31]. L1 elements are non-long terminal repeat (LTR) retrotransposons, containing an internal promoter in the 5′ untranslated region (5′UTR) that initiates transcription followed by two long open reading frames, ORF1 and ORF2, required for retrotransposition [32]. The L1 retrotransposition machinery is not only used for L1 mobilization itself, but also assists in the retrotransposition of Alu retroelements [33]. Alu elements modulate gene transcription by binding several transcription factors [34]. L1 has been reported to affect gene expression by introducing polyadenylation signals, although this observation is partially speculative [35]. Given these important regulatory functions, random point mutations to Alu and L1 elements could potentially introduce novel gene-regulatory functions, as observed previously for alternative splice site formation [30]. To better understand the fine regulation of L1 and Alu retroelements induced by benzene exposure, we focused on repetitive elements subfamilies of different evolutionary age [15]. Repetitive element subfamilies are individually regulated and have different CpG and DNA methylation frequencies; therefore, they might have different sensitivities to environmental exposures.

The present results showed that increased expression of Alu-Y and Alu-J was associated with an increased internal dose of benzene, estimated by way of its biomarkers BEN-U and *tt*-MA. Additionally, increased L1-5′UTR expression was associated with *tt*-MA levels (Table 4). The finding might indicate that transcriptionally active retrotransposons, which are a small subset of the total genomic elements of the same family [36], respond to these specific exposures. However, it is important to observe that, while BEN-U is a specific biomarker of benzene exposure, *t,t*-MA is not, as it may be also present as biotransformation product of food preservative agents sorbic acid/sorbates. This additional source makes critical the use of *t,t*-MA as biomarker of low occupational exposures, such as those observed in the present study. The result reported in Table 1, showing no difference in *t,t*-MA comparing petrol station workers and controls, confirms this issue. Additionally, no increased expression of repetitive elements associated with BEN-A or SPMA levels was found. All this considering, the observed modifications of retroelements expression in association with different markers is difficult to explain and might be due to differences between internal and external dose and/or between different metabolic pathways involving the different markers or even associated with exposures different from those here investigated.

Regarding repetitive element subfamily methylation, only L1-Pa5 was hypomethylated in petrol station exposed workers compared to controls (Table 2). However, L1-Pa5 methylation was not associated with benzene exposure or its biomarkers (Table 3 and Table 4). The decreased methylation of L1-Pa5 may be associated with long term occupational exposures to petrol exhaust, but not with daily exposures. Interestingly, trends of decreased L1-HS methylation, and more generally, of methylation at any investigated site, were observed with increased levels of *tt*-MA, although none of these trends was statistically significant (Table 4). This result is in line with our previous findings of a negative association between Alu and L1 methylation and *tt*-MA [9]. The observed decreased DNA methylation may be explained by oxidative stress associated with the formation of *t,t*-muconaldehydes, metabolic precursors of *tt*-MA [37]. Interestingly, a negative association between Alu-YD6 and L1-Ta methylation and MTBE-U was found (Table 4). Considering that MTBE-U is a reliable marker for assessing urban traffic and petrol vapours exposure [2], the observed association between DNA methylation and MTBE might be suggestive of a specific effect exerted by petrol vapors and/or urban traffic components (such as particulate matter) other than benzene.

Previously described global repetitive element methylation changes [8,9] might be caused by retroelement subfamilies that were not investigated in the present study. Moreover, the observed DNA methylation changes might represent the average expression level of a group of repetitive elements where only a subset of elements has significant methylation and expression changes, effectively diluting the strength of the effect. It is worth noting that retroelement derepression uncoupled from DNA hypomethylation has recently been observed by our group in human leukemia and hematopoietic stem cells in response to hydroquinone, an important benzene-derived metabolite [23]. It thus seems likely that the same type of benzene-responsive epigenetic switch observed in vitro may be at work in human subjects. The mechanism of this response may involve transactivator proteins binding and transcriptionally activating Alu elements in human cells [36]. The concentration and/or activity of these transactivators could be influenced by benzene metabolites, possibly through the enhancement of reactive oxygen species [38].

The third level of investigation in our study addressing histone H3K9me3 modification, which was reported previously to suppress transcription of Alu elements [22]. Aberrant histone modifications have been reported in several cancers [39] and following exposure to environmental exposures both in in vitro models [40] and in exposed subjects [41]. In this study, the extent of histone H3K9me3 modification in human subjects occupationally exposed to petrol vapors benzene was not associated with airborne benzene levels or with urinary metabolites. The only study evaluating whether benzene exposure alters levels of global histone methylation or acetylation was conducted on murine tissues [42] and, in agreement with our results, reported no effect of exposure on any form of histone modification.

Major limitations of this study are associated with the low occupational exposure of petrol station workers; the complexity of petrol-related chemical exposure; and several factors, other than occupational exposure, potentially impacting epigenetic and transcriptional markers. Regarding the first point it is worth to note that median exposure to benzene is more than one order of magnitude lower than occupational exposure limits, and this low exposure may reduce the ability of epigenetic and transcriptional factors to show consistent and significant modifications. Moreover, being petrol a mixture of several chemicals, we are not able to exclude that the observed effects are due to hydrocarbons/pollutants other than benzene, either as single compound or as a mixture. In addition, study subjects, recruited in real life environment, are characterized by different health status and are exposed to a multitude of non-occupational related factors (such as diet and drugs) that may confound the relationship between epigenetic modifications and occupational exposure; although in the present study a big effort to control such factors was done (recruiting healthy subjects, registering health status and drug consumption, measuring health-related blood parameters, restricting diet) we acknowledge that there may be other confounders, not considered in the present investigation.

## 5. Conclusions

Overall, these findings only partially support previous observation linking benzene exposure with global DNA hypomethylation and underscore the complexity of retroelement regulation in response to benzene and other petrol-related chemicals. These results provide a rationale for further molecular studies to evaluate comprehensively the relationships between environmental exposure to benzene and epigenetic changes occurring at retroelements, with the long-term aim to clarify how these molecular events are related to disease pathology. A better understanding of epigenetic alterations occurring after exposure to occupational and environmental factors with a well-established role as carcinogens, would provide important insights into the epigenetic mechanisms underlying carcinogenesis. Although several studies have demonstrated a mechanistic link between DNA hypomethylation and genetic changes commonly observed in cancer, only observational reports on carcinogen-induced DNA hypomethylation are available, but a clear demonstration of the mechanism linking loss of DNA methylation and cancer development is still lacking [43,44]. The investigation of the epigenetic mechanisms by which epigenetic carcinogens promote cancer development will probably be, in the future, incorporated in cancer hazard assessment, but a lot of work is still needed in order to reach this important goal.

## Figures and Tables

**Table 1:** Summary of general characteristics, markers of exposure and health status in petrol station workers and control subjects.

Personal Characteristic	Statistics	Petrol Station Workers (*N* = 89)	Controls (*N* = 90)	*p*
Age (years)	mean ± SD	44 ± 11	44 ± 11	0.9671
Sex (male)	N (%)	89 (100%)	90 (100%)	-
BMI (kg/m^2^)	mean ± SD	25.9 ± 3.1	26.2 ± 3.4	0.6000
Cotinine				
>100 μg/L	N (%)	40 (45%)	36 (40%)	0.5034
≤100 μg/L	N (%)	49 (55%)	54 (60%)	
Cotinine in smokers (µg/L)	Median (10th–90th)	1948 (984–2933)	1691 (462–2856)	0.4700
Creatinine * (g/L)	median (10th–90th)	1.66 (0.92–2.40)	1.54 (0.92–2.35)	0.1670
**Exposure assessment**				
BEN-A (μg/m^3^)	median (10th–90th) minimum–maximum	59 (10–203) 3–3246	4 (2–112) 1–48	<0.0001
MTBE-A (μg/m^3^)	median (10th–90th) minimum–maximum	408 (32–1905) 2–57616	3.5 (1.7–15.3) <0.8–528	<0.0001
BEN-U * (ng/L)	median (10th–90th) minimum–maximum	382 (114–2201) 71–4702	122 (72–948) 54–4407	<0.0001
*tt*-MA * (µg/L)	median (10th–90th) minimum–maximum	86 (22–185) <20–505	63 (25–184) < 20–292	0.3324
SPMA * (µg/L)	median (10th–90th) minimum–maximum	0.17 (<0.1–1.01) <0.1–2.19	<0.1 (<0.1–0.68) <0.1–0.68	0.0002
MTBE-U * (ng/L)	median (10th–90th) minimum–maximum	232 (85–751) 14–1733	58 (22–109) 8–158	<0.0001

* Geometric mean of three measures: baseline, start-shift and end-shift levels.

**Table 2:** Differences in gene expression, DNA methylation, and histone modification in peripheral blood lymphocytes between petrol station workers and control subjects.

**Variable**	**β**	**95% CI**		***p***
Gene expression (ΔCT)				
*Alu*-Y	0.05	−0.12	0.22	0.53
*Alu*-J	0.13	−0.06	0.32	0.19
L1-5U’TR	0.02	−0.13	0.17	0.77
L1-ORF1	0.08	−0.06	0.22	0.25
	**β**	**95% CI**		***p***
Methylation (%5mC)				
*Alu*-YB8	0.05	−0.42	0.53	0.82
*Alu*-YD6	−0.09	−0.46	0.27	0.61
*Alu*-SX	0.11	−0.17	0.38	0.45
L1-HS	0.13	−0.28	0.54	0.54
L1-Pa2	0.16	−0.33	0.66	0.51
L1-Pa5	−1.02	−2.04	0.01	0.05
L1-Ta	−0.08	−0.45	0.29	0.67
	***e*^β^**	**95% CI**		***p***
Histone modification (ng/µL)				
H3K9me3 *	1.09	−1.04	3.23	0.30

β represents the mean differences in relative gene expression or % methylation between petrol station workers and control subjects. It is expressed as ΔCT for relative gene expression and as %5mC for methylation markers. e^β^ = exp(β) represents the geometric mean difference in H3K9me3 between petrol station workers and control subjects. * H3K9me3 concentrations were log-transformed. All models are adjusted for age, BMI, cotinine and % lymphocytes. Models for methylation data are adjusted for run, position, plate and their interaction term. Linear regression models for histone modification are adjusted based on a standard curve. Significant *p*-values are in bold.

**Table 3:** Association between gene expression, DNA methylation, and histone modification in peripheral blood lymphocytes and personal exposure to airborne benzene and MTBE in all study subjects (*n* = 179 subjects).

**Variable**	**BEN-A**				**MTBE-A**			
	**β_90-10_**	**95% CI**		***p***	**β_90-10_**	**95% CI**		***p***
Gene expression (ΔCT)								
*Alu*-Y	−0.010	−0.051	0.031	0.6392	−0.004	−0.024	0.015	0.6787
*Alu*-J	−0.011	−0.056	0.035	0.6404	−0.006	−0.028	0.016	0.5929
L1-5′UTR	0.020	−0.015	0.056	0.2654	0.009	−0.008	0.026	0.2843
L1-ORF1	0.029	−0.004	0.063	0.0871	0.013	−0.003	0.029	0.1167
	**β_90-10_**	**95% CI**		***p***	**β_90-10_**	**95% CI**		***p***
Methylation (%5mC)								
*Alu*-YB8	0.025	−0.089	0.138	0.6728	0.015	−0.040	0.069	0.5955
*Alu*-YD6	0.045	−0.042	0.133	0.3098	0.029	−0.013	0.071	0.1727
*Alu*-SX	0.039	−0.025	0.102	0.2342	0.014	−0.016	0.045	0.3554
L1-HS	0.026	−0.073	0.125	0.6062	0.017	−0.030	0.064	0.4841
L1-Pa2	0.048	−0.068	0.165	0.4186	0.026	−0.029	0.082	0.3554
L1-Pa5	0.060	−0.194	0.313	0.6456	0.039	−0.081	0.160	0.5237
L1-Ta	0.017	−0.073	0.106	0.7176	0.013	−0.030	0.056	0.5531
	**Δ%_90-10_**	**95% CI**		***p***	**Δ%_90-10_**	**95% CI**		***p***
Histone modification (%)								
H3K9me3 *	0.545	−3.423	4.677	0.7917	0.213	−1.698	2.161	0.8291

* H3K9me3 concentrations were log-transformed. β_90-10_ and Δ%**_90-10_** = (e^β^_90-10_ − 1) * 100 expressing the change in gene expression (ΔCT), methylation (%5mC) and histone H3K9me3 (%) associated with an increase in airborne exposure equal to the difference between the 90th and 10th percentile of the exposure distribution.

**Table 4:** Association between gene expression, DNA methylation, and histone modification in peripheral blood lymphocytes and urinary biomarkers of exposure in all study subjects (*n* = 179 subjects).

**Variable**	**BEN-U**				***tt*-MA**				**SPMA**				**MTBE-U**			
	**β_90-10_**	**95% CI**		***p***	**β_90-10_**	**95% CI**		***P***	**β_90-10_**	**95% CI**		***p***	**β_90-10_**	**95% CI**		***p***
Gene expression (ΔCT)																
*Alu*-Y	0.290	0.046	0.533	**0.0210**	0.410	0.191	0.628	**0.0003**	0.131	−0.114	0.376	0.2953	0.039	−0.076	0.154	0.5082
*Alu*-J	0.458	0.191	0.724	**0.0010**	0.331	0.082	0.580	**0.0100**	0.154	−0.119	0.427	0.2710	0.066	−0.063	0.194	0.3169
L1-5’UTR	0.031	−0.185	0.246	0.7811	0.270	0.076	0.463	**0.0070**	−0.034	−0.248	0.179	0.7536	0.046	−0.055	0.146	0.3745
L1-ORF1	0.092	−0.112	0.296	0.3779	0.099	−0.089	0.286	0.3030	−0.171	−0.372	0.030	0.0977	0.034	−0.062	0.129	0.4892
	**β_90-10_**	**95% CI**		***p***	**β_90-10_**	**95% CI**		***p***	**β_90-10_**	**95% CI**		***p***	**β_90-10_**	**95% CI**		***p***
Methylation (%5mC)																
*Alu*-YB8	0.218	−0.489	0.924	0.5467	−0.228	−0.847	0.391	0.4722	0.289	−0.455	1.033	0.4474	0.049	−0.273	0.370	0.7676
*Alu*-YD6	−0.203	−0.749	0.343	0.4670	0.061	−0.419	0.542	0.8035	−0.222	−0.794	0.351	0.4486	−0.257	−0.502	−0.012	**0.0411**
*Alu*-SX	0.005	−0.406	0.415	0.9820	−0.136	−0.492	0.220	0.4545	0.406	−0.017	0.829	0.0616	−0.037	−0.218	0.144	0.6876
L1-HS	0.081	−0.536	0.698	0.7975	−0.515	−1.052	0.022	0.0620	−0.137	−0.784	0.510	0.6787	−0.220	−0.498	0.058	0.1232
L1-Pa2	−0.364	−1.094	0.367	0.3305	−0.166	−0.810	0.478	0.6137	−0.224	−0.991	0.542	0.5671	0.132	−0.198	0.462	0.4333
L1-Pa5	0.071	−1.527	1.669	0.9307	−0.061	−1.440	1.318	0.9312	0.303	−1.344	1.950	0.7190	−0.407	−1.117	0.303	0.2625
L1-Ta	−0.096	−0.656	0.464	0.7377	−0.126	−0.623	0.371	0.6191	−0.086	−0.680	0.508	0.7767	−0.279	−0.529	−0.030	**0.0298**
	**Δ%_90-10_**	**95% CI**		***p***	**Δ%_90-10_**	**95% CI**		***p***	**Δ%_90-10_**	**95% CI**		***P***	**Δ%_90-10_**	**95% CI**		***p***
Histone modification (%)																
H3K9me3 *	−1.799	−23.084	25.376	*0.8844*	−9.209	−28.292	14.952	0.4235	9.348	−13.92	38.907	0.4652	0.825	−10.017	12.974	0.8876

* H3K9me3 concentrations were log-transformed. β _90-10_ and Δ%_90-10_ = (e^β^_90-10_ − 1) * 100 expressing the change in gene expression (ΔCT), methylation (%5mC) and histone H3K9me3 (%) associated with an increase in urinary exposure equal to the difference between the 90th and 10th percentile of the exposure distribution. Significant *p*-values are in bold.

## Supplementary Materials

The following are available online at http://www.mdpi.com/1660-4601/15/4/735/s1, Figure S1: Primers for gene expression real-time PCR, and methylation, including PCR conditions. Table S1: Summary of health status, including work-related symptoms, present and previous diseases, drug assumption, blood cell count, liver enzymes, triglycerides, cholesterol and C-reactive protein (CRP) in petrol station workers and control subjects. Only reported symptoms/diseases/drugs are included.
