# Supplementary material for: Epigenetic and Transcriptional Modifications in Repetitive Elements in Petrol Station Workers Exposed to Benzene and MTBE
Source: Int J Environ Res Public Health. 2018 Apr 12;15(4):735. doi: 10.3390/ijerph15040735 (PMC5923777; doi:10.3390/ijerph15040735)
Supplement: Supplementary file 1 [file ijerph-15-00735-s001.zip › Supplementary Figure 1 Rota et al.pdf]

Supplementary Figure S1. Primers for gene expression real-time PCR, and methylation, including PCR conditions.

Gene expression eal-time PCR primers			
**Family**	**qPCR Target RNAs**	**Forward primer (5' to 3')**	**Reverse primer (5' to 3')**
Alu	*Alu*Y	CAAGGTGAAACCCCGTCTCTAC	CCATTCTCCTGCCTCAGCC
	*Alu*J	CATAGCGAGACCCCGTCTCTAC	CGATCCTCCTGCCTCAGC
LINE-1	L1 5'UTR	CTCAAGTGGGTCCCTGACTCC	TCTGTTGGAATACCCTGCCG
	L1 ORF1	GGACATTCAAACCAAAGGCAA	ATCAGCTCCTTTAAGCACTTCTCTG
Housekeeping gene	B2M	GGCTATCCAGCGTACTCCAAAG	TGGATGAAACCCAGACACATAGC

Methylation primers					
**Family**	**Assay**	**Forward Primer (5' to 3')**	**Reverse Primer (5' to 3')**	**Sequencing Primer (5' to 3')**	**Annealing temperature (°C)**
Alu	**AluYb8**	Biotin-AGATTATTTTGGTTAATAAG	AACTACYAACTACAATAAC	AATAACTAAAACTACAAAC	53.9
	**AluSx**	Biotin-TTTTTATTAAAAATATAAAAATT	CCCAAACTAAAATACAATAA	AATAACTAAAATTACAAAC	50.0
LINE-1	**L1Pa5**	TTAGTTAAGGGAAGAGGGGATAAA	Biotin-ATAAACATAAAACCCTCTAAACCAAACA	TTAGTTAAGGGAAGA	40.0
	**L1Pa2**	TTAGATAGTGGGYGTAGGTTAGTGGGT	Biotin-CCTCCRAACCAAATATAAAATATAATCT	GAGTTAAAGAAAGGG	55.0
	**L1Hs**	TTTTGAGTTAGGTGTGGGATATA	Biotin-AAAATCAAAAAATTCCCTTTC	AGTTAGGTGTGGGATATAGT	56.3
