# Supplementary material for: Epigenetic and Transcriptional Modifications in Repetitive Elements in Petrol Station Workers Exposed to Benzene and MTBE
Source: Int J Environ Res Public Health. 2018 Apr 12;15(4):735. doi: 10.3390/ijerph15040735 (PMC5923777; doi:10.3390/ijerph15040735)
Supplement: Supplementary file 1 [file ijerph-15-00735-s001.zip › Supplementary Table 1 Rota et al.pdf]

Supplementary Table S1. Summary of health status, including work-related symptoms, present and previous diseases, drug assumption, blood cell count, liver enzymes, triglycerides, cholesterol and C-reactive protein (CRP) in petrol station workers and control subjects. Only reported symptoms/diseases/drugs are included.

	**Statistics**	**Petrol station workers (N = 89)**	**Controls (N=90)**	**P-value**
**Work-related symptoms**	Yes N (%)	18 (20.2)	12 (13.3)	0.217
Headache	N (%)	5 (5.6)	4 (4.4)	0.719
Throw irritation	N (%)	4 (4.5)	4 (4.4)	0.987
Eye irritation	N (%)	9 (10.1)	4 (4.4)	0.144
Cough	N (%)	6 (6.7)	3 (3.3)	0.297
Skin irritation	N (%)	3 (3.4)	3 (3.3)	0.989
**Diseases (last 30 days)**	Yes N (%)	5 (5.5)	5 (5.6)	0.985
Cold	N (%)	4 (4.5)	2 (2.2)	0.398
Hepatitis	N (%)	1 (1.1)	0	0.313
Herpes simplex	N (%)	0	3 (3.3)	0.082
**Previous diseases (last six months)**	Yes N (%)	28 (31.4)	19 (21.1)	0.116
Cold	N (%)	24 (26.9)	16 (17.8)	0.140
Hepatitis	N (%)	1 (1.1)	1	0.994
Herpes simplex	N (%)	4 (4.5)	7 (7.8)	0.360
**Drugs (present)**	Yes N (%)	4 (4.5)	3 (3.3)	0.987
Oral antidiabetics	N (%)	3 (3.4)	2 (2.2)	0.641
Penicillin	N (%)	0	1 (1.1)	0.319
Glucocorticoids	N (%)	1 (1.1)	0	0.313
Heparin	N (%)	0	1 (1.1)	0.319
**Previous drugs (last 30 days)**	Yes N (%)	12 (13.5)	4 (4.4)	0.034
Oral antidiabetics	N (%)	2 (2.2)	1 (1.1)	0.554
Penicillin	N (%)	1 (1.1)	2 (2.2)	0.567
Glucocorticoids	N (%)	3 (3.3)	1 (1.1)	0.306
heparin	N (%)	1 (1.1)	0	0.313
UV radiation	N (%)	4 (4.5)	0	0.042
**Blood test**				
White cells (N/L)	mean ± SD	7.64 ± 1.84	7.01 ± 1.75	0.0198
Red cells (N/L)	mean ± SD	5.09 ± 0.41	4.98 ± 0.36	0.0428
Hemoglobin (g/dL)	mean ± SD	15.0 ± 1.1	14.9 ± 1.1	0.5552
Hematocrit (%)	mean ± SD	43.9 ± 2.9	43.3 ± 2.6	0.1758
MCV (fL)	mean ± SD	86.0 ± 5.7	86.9 ± 5.7	0.3103
Platelet (N/L)	mean ± SD	224.1 ± 56.1	220.6 ± 54.0	0.6742
AST (IU/L)	median (10th-90th)	24 (19-39)	25 (19-39)	0.766
ALT(IU/L)	median (10th-90th)	26 (15-47)	26 (16-63)	0.3028
GGT (IU/L)	median (10th-90th)	24 (15-63)	24 (14.5-55)	0.4731
Triglycerides (mg/dL)	median (10th-90th)	130 (74-301)	113 (61-273)	0.0172
Cholesterol (mg/dL)	mean ± SD	194 ± 39	188 ± 39	0.3313
HDL cholesterol (mg/dL)	mean ± SD	48 ± 13	49 ± 12	0.9133
LDL cholesterol (mg/dL)	mean ± SD	114 ± 35	113 ± 34	0.8239
CRP (mg/L)	median (10th-90th)	0.12 (0.03-0.52)	0.12 (0.03-0.34)	0.5631
