# Supplementary material for: Epigenetic and Transcriptional Modifications in Repetitive Elements in Petrol Station Workers Exposed to Benzene and MTBE
Source: Int J Environ Res Public Health. 2018 Apr 12;15(4):735. doi: 10.3390/ijerph15040735 (PMC5923777; doi:10.3390/ijerph15040735)
Supplement: Supplementary file 1 [file Review Report Form_SF-reviewer 2 .pdf › Review Report Form_SF-reviewer 2 .pdf]

Review Report Form 2

English language and style

( ) Extensive editing of English language and style required

(x) Moderate English changes required

( ) English language and style are fine/minor spell check required

( ) I don't feel qualified to judge about the English language and style

	Yes	Can be improved	Must be improved	Not applicable
Does the introduction provide sufficient background and include all relevant references?	( )	(x)	( )	( )
Is the research design appropriate?	(x)	( )	( )	( )
Are the methods adequately described?	(x)	( )	( )	( )
Are the results clearly presented?	( )	(x)	( )	( )
Are the conclusions supported by the results?	(x)	( )	( )	( )

Comments and Suggestions for Authors

This is a well designed study of the association between exposure to benzene and MTBE in an occupational setting, and epigenetic alterations in surrogate tissues (blood).

Thank you for your favorable comment

Overall, the authors have not presented a great deal of new information relative to their previous publications. This reviewer suggests that the authors highlight the lack of an association between several of the endpoints measured, and MTBE exposure, rather than making an unsubstantiated claim that the methylation profile is related to hematopoietic malignancies.

Thank you, we have taken this suggestion into account and introduced words of caution in several parts of the text. In particular in line 26, last sentence of the abstract; line 304-305, line 342-346 and 397 in the Discussion.

The authors cite reports of an association between exposure and methylation, but all but assume the reader is familiar with the body of evidence of benzene-related tumorigenesis, and/or methylation marks and cancer. Further, the authors should note that construction workers are likely exposed to exhaust fumes at a higher level compared to the other occupations listed for the control subjects. The authors may consider removed construction workers to see if the differences between the controls and the exposed groups are more substantial.

We checked the jobs of control subjects and found that the word “construction workers” was used as synonymous of bricklayers; we have now changed the word. Moreover, we checked the questionnaires and exclude that these subjects were occupationally exposed to diesel exhaust fumes and emissions from vehicles or to other organic solvents that could interfere with our measurements; to make it clear we added a sentence in the text. Accordingly we did not performed a new statistical analysis.

Overall, several issues should be addressed to improve the paper before it is accepted for publication.

Generally, change gender to sex.

Thank you, we have changed as suggested.

Abstract:

The authors refer to the authors should state more clearly what histone methylation profile was measured; to say "methylation [...] of histones" is not informative to the reader.

The information has been added.

The exposure level to the various chemicals should also include a specified duration or rate.

The information has been added.

Introduction:

Page 2, Lines 45-48: the authors surmise that the global hypomethylation that has been demonstration in relation to benzene exposure may contribute to hematotoxicity; however this supposition is not substantiated. If the authors want to make this argument, further evidence of the

role of hypomethylation in hematotoxicity is warranted to "connect the dots" between the attributes.

In agreement with reviewer’s comment, we added the following paragraph:

Lines 49-52: “Despite the mentioned data, the evidences in vivo are still limited and mainly reporting associations more than a causal link between exposure, epigenetic variations and cancer development, which would request the use of prospective studies in order to avoid reverse causation.”

Page 2, Lines 72-79: The objective of the study is nicely stated, but is missing the matrix in which the molecular measures were made - urine, plasma? Final sentence - suggest refraining from stating conclusions in the introduction.

Thank you, the matrix has been added.

Generally, the authors should make additional mention regarding why a better understanding of epigenetic alterations is important in environmental toxicology and cancer; see recent reviews by G. Chappell and/or Z. Herceg.

We thank the reviewer for pointing out this important aspect. We integrated in the discussion the following paragraph:

Lines 391-400: “A better understanding of epigenetic alterations occurring after exposure to occupational and environmental factors with a well-established role as carcinogens, would provide important insights into the epigenetic mechanisms underlying carcinogenesis. Although several studies have demonstrated a mechanistic link between DNA hypomethylation and genetic changes commonly observed in cancer, only observational reports on carcinogen-induced DNA hypomethylation are available, but a clear demonstration of the mechanism linking loss of DNA methylation and cancer development is still lacking. The investigation of the epigenetic mechanisms by which epigenetic carcinogens promote cancer development will probably be, in the future, incorporated in cancer hazard assessment but a lot of work is still needed in order to reach this important goal.”

Methods:

Line 88: late=last

Done.

What is the method for collecting data on personal exposure levels? this should be stated earlier in the paragraph, at first mention of the collection of the measurements. Even if referencing a previous publication, a brief statement/description of the method should be included.

The information is reported at the beginning of the paragraph “Air and urinary exposure assessment”.

For RTPCR, the 2–ΔΔCt method of Livak and Schmittgen, 2001 is recommended unless the authors can justify the formula currently applied.

When we selected the statistical strategies for analysing our data, we carefully evaluated whether our data could be analyzed as 2^-dCt. However, the distribution of expression data, when presented as 2^-dCt, appeared highly skewed and not consistent with the normal Gaussian distribution. At the opposite –dCt data were fairly symmetric and satisfactorily approximated by a Gaussian distribution. Because the statistical methods we used assumed that the data followed a Gaussian distribution, in the revised manuscript we still present the analyses based on –dCt.

To further understand why we used -dCT in our analyses, please consider the following example. The asymmetrical distribution (with the maximum values shifted to the left) of our dependent variable (expression) measured as 2^(-dCT) is represented in Fig. 1. In the presence of such skewed data, it is standard statistical practice to use a logarithmic data transformation (Fig. 2), which in the case in discussion was equivalent to –dCt.

As shown in Table 1, the only difference between 2-dCT and -dCT values is the scale of measurement. On the 2-dCT scale, values greater than 1 represent an increased expression, whereas values less than 1 represent a decrease of the expression. Conversely, if we consider –dCT, values greater than 0 representing an increase of the expression while values below 0 represent a decrease of the expression.

Fig.1

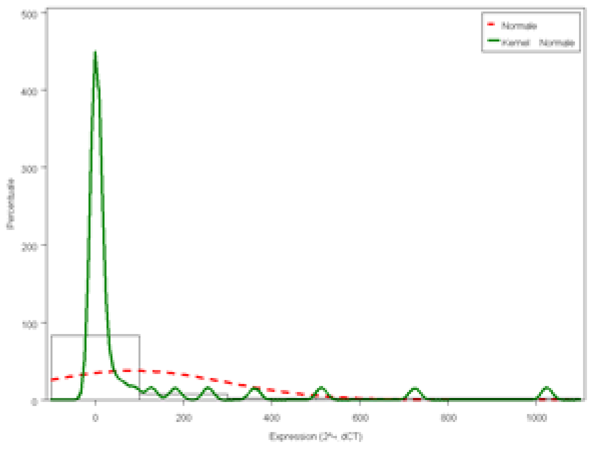

Fig.2

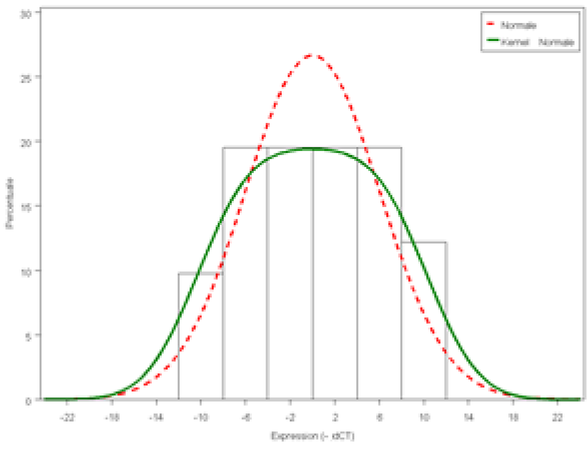

Table 1

Before Transformation

**2^{-dCT}**	0.001	0.004	0.016	0.063	0.250	1	4	16	64	256	1024

After Transformation

**- dCT**	-10	-8	-6	-4	-2	0	2	4	6	8	10

In addition, the use of dCt instead of ddCt is also justifiable as relative quantification (RQ)can be performed either with 2^(- ddCt) method (which is the standard method for investigating differences among groups) or 2^(-dCt) method (see for example "Relative quantification" by Michael W. Pfaffl in: Real-time PCR. Published by International University Line (Editor: T. Dorak), p. 66). Both methods were also explained and discussed in the referenced paper mentioned by reviewer 1 (Livak and Schmittgen 2001). The use of the dCt allows to consider the variable as continuous in order to maintain the variability of the variable and the statistical power of the correlation.

Results:

Table 2 - is beta simply change, as in delta? OR is this fold change over the average of the control group? This should be made more clear in the tables in the results section.

We are thankful to the reviewer for his/her suggestion. We corrected Table 2, to take this into account.

Same question for beta90-10; better clarification of what is meant by 'change in percent methylation associated with an increase in urinary metabolite concentration ...' is needed. This is not clear enough in the methods, or the results, for the reader to judge the confidence in any associations presented.

We thank reviewer for comment. We rewrote the sentence to be more clear as below:

$\beta_{90\text{-}10}$ expresses the change in methylation (%5mC) for an increase equal to the difference between the 90th and 10th percentile of the urinary metabolite distribution, whereas not standardized β coefficient measures the change in outcome variable for one unit increase in predictor variable. We corrected also Table 3 and Table 4 to take this into account.

Discussion:

On line 299, the authors state:

"The main hypothesis of our study was that molecular events occurring at retroelements might play a key role in early leukemogenesis." There is nearly no information throughout the manuscript that addresses this hypothesis. The authors make a large leap from associative analyses of exposure metrics and molecular signatures, all the way to disease outcome. If the authors state this hypothesis to be the main point of their study, then further analyses are necessary. What is the incidence of hematopoietic cancers in the study subjects, workers or controls? What is the incidence of any other cancers? Were molecular markers of epigenetic change related to any other biomarker that is an early indicator of disease? Without such results, the hypothesis is un-addressed. It is understood that such information may not be available for the participants, but some discussion surrounding the relevance of the epigenetic markers studied, as well as the general relationship between exposure to the chemicals and cancer, should be discussed with appropriate references.

As the reviewer pointed out, the hypothesis of the present study is not the one we stated in the paper. We therefore changed the sentence as "The main hypothesis of our study was that occupational exposure to benzene might be associated to molecular events occurring at retroelements, which are relevant as they might potentially play a key role in early leukemogenesis."
Follow-up information are not available for the study participants, and given the limited number of subjects, we would not aspect to observe a sufficient number of cases to have an adequate statistical power.

Additionally, we introduced a sentence citing the reviews of G. Chappell and Z. Herceg, as useful documents to get an overview on epigenetic alterations in environmental chemical carcinogenesis.

A good discussion point would be surrounding the differences in the epigenetic profile between two chemicals that are classified differently by IARC, and the reasons behind that hazard classification. Are the chemicals thought to cause cancer by a different mode of action, or is the mode of action unknown? This should be discussed so that the reader has an understanding of how and why the epigenetic marks may be incorporated into human hazard assessment.

This very interesting suggestion would require a wide dissertation and additional work that goes behind the scope of the present paper and may feed further development of our research.
